# Analytic Study of Complex Fractional Tsallis’ Entropy with Applications in CNNs

**DOI:** 10.3390/e20100722

**Published:** 2018-09-20

**Authors:** Rabha W. Ibrahim, Maslina Darus

**Affiliations:** 1 Faculty of Computer Science and Information Technology, University of Malaya, Kuala Lumpur 50603, Malaysia; 2 School of Mathematical Sciences, Faculty of Sciences and Technology, Universiti Kebangsaan Malaysia, Bangi 43600, Selangor, Malaysia

**Keywords:** fractional calculus, fractional operator, fractional entropy, CNNs, analytic function, unit disk

## Abstract

In this paper, we study Tsallis’ fractional entropy (TFE) in a complex domain by applying the definition of the complex probability functions. We study the upper and lower bounds of TFE based on some special functions. Moreover, applications in complex neural networks (CNNs) are illustrated to recognize the accuracy of CNNs.

## 1. Introduction

A strategic amount in information theory is entropy. Entropy measures the amount of uncertainty appearing in the assessment of a random variable or the outcome of a random process. In 1988, Tsallis [1] presented the nonadditive entropy, aiming at a generalization of Boltzmann–Gibbs (BG) statistical mechanics. The purpose of this generalization is to study complex systems. Its applications appeared in many fields, such as thermodynamics, chaos, artificial neural networks, image processing, complex systems, information theory, etc. (see [2,3,4,5,6,7,8,9,10,11,12,13,14,15]).

The scheme of the axioms of probability theory placed in 1933 by Kolmogorov can be extended to include the imaginary set of numbers and this by accumulation to his original five axioms. Later, an additional three axioms were given in [16]. Consequently, the complex probability domain is defined by the sum of the real set $S_{R}$ with its corresponding real probability and the imaginary $S_{M}$ with its corresponding imaginary probability. In general, the advantages of complex probability theory are that it is considered a supplementary dimension (imaginary part) to the event appearing in the real dimension laboratory (real part). It represents physical quantities of complex networks in terms of currents, complex potentials and impedance. Moreover, it fulfills luck and chance in $S_{R}$ substituted by total determinism in a complex domain. Finally, it extends many well-known concepts of the traditional probability theory, such as expectation and variance, to the complex probability theory with more accuracy in applications. One of the important applications of complex probability theory is in realistic quantum mechanics [17]; for example, the two slit experiment where a source releases a single particle, which moves to a wall with two slits and is spotted at position χ on a shelter placed behind the wall. The typical argument that an interference design on the shelter infers that the particle did not either drive through one slit or the other is ultimately an argument in probability theory such that $P\left(\chi \right)=P{\left(\chi \right)}_{1}+P{\left(\chi \right)}_{2},$ where $P{\left(\chi \right)}_{1}$ and $P{\left(\chi \right)}_{2}$ are the probability via the first and second slit, respectively, which is a critical process. This process leads to the use of complex probability theory.

**Figure 1:**
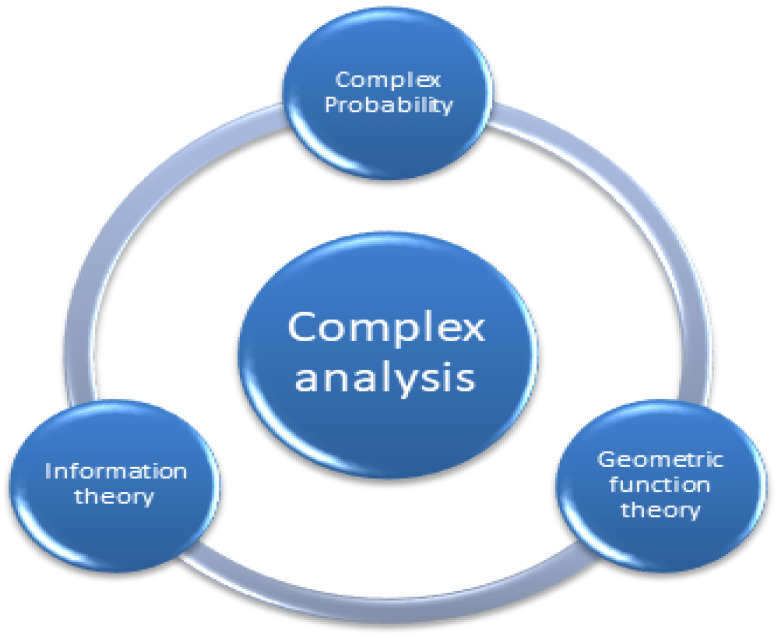
The connection of the main objectives of this research.

Recently, Abou Jaoude [18] extended Shannon’s information theory by using the complex probability. The author calculated the magnitude of the chaotic factor, the channel capacities in the probability and the degree of knowledge. In general, complex probability leads to better information for all processes compared to the classical probability [19,20]. Figure 1 shows the relation between complex analysis and information theory.

Our investigation is based on the concept of complex probability to extend the idea of Tsallis’ fractional entropy (TFE). The study of the technique delivered by using the approximation theory of special functions of complex variables was useful in information theory. We introduce the upper and lower bound of TFE. Sharpness is discussed as well in the sequel.

## 2. Results

Let *A* be an event in a complex domain $S_{C}$. The real and imaginary terms of the complex probability function (CPF):

$$P_{c}\left(z\right)=P_{r}\left(x,y\right)+P_{m}\left(x,y\right)$$

where the argument z=x+iy, and $P_{r}$ and $P_{m}$ are the real probability and the imaginary probability in the real set $S_{R}$ and imaginary set $S_{M}$, respectively. Following Axiom 7 in [16], we have:

(1) $$P_{c}\left(z\right)=P_{r}\left(x,y\right)+i\left(1-P_{r}\left(x,y\right)\right),$$

such that z=x+iy with ${\left|z\right|}^{2}=P_{r}^{2}+{\left(P_{m}/i\right)}^{2}$ and $P_{m}=i\left(1-P_{r}\right);$ hence, $P_{c}$ is always equal to one. Abou Jaoude et al. [19] inferred that z∈U={z∈C:|z|<1} (the open unit disk).

Tsallis presented an entropic formalization characterized by an index γ, which implies a non-extensive statistics. TFE ($T_{\gamma }$) is the basis of the so-known non-extensive statistical mechanics, which modifies the Boltzmann–Gibbs theory. Tsallis statistics has been used in various fields such as applied mathematics, physics, biology, chemistry, computer science, information theory, engineering, medicine, economics, business, geophysics, etc. Since we study the analytic properties of TFE, therefore, we focus on the continuous formula. The general continuous form of this entropy is given by:

$$T_{\gamma }\left[P\right]=\frac{1}{\gamma -1}\left(1-\int_{x}{\left(P\left(x\right)\right)}^{\gamma }dx\right),\;\gamma \neq 1.$$

By applying the concept of CPF in Equation (1), we extend TFE into complex values as follows (CTFE):

(2) $$T_{\gamma }\left[P_{c}\right]=\frac{1}{\gamma -1}\left(1-\int_{S_{C}}{\left(P_{c}\left(z\right)\right)}^{\gamma }\;dz\right).$$

For a special domain $S_{C}=U,$ we have:

(3) $$T_{\gamma }{\left[P_{c}\right]}_{U}=\frac{1}{\gamma -1}\left(1-\int_{U}{\left(P_{c}\left(z\right)\right)}^{\gamma }\;dz\right).$$

For the analytic study, we shall use the definition:

(4) $$T_{\gamma }\left(z\right):=\left(\gamma -1\right)T_{\gamma }{\left[P_{c}\right]}_{U}=1-\int_{0}^{z}{\left(P_{c}\left(w\right)\right)}^{\gamma }\;dw,\;z\in U,$$

where $P_{c}$ is analytic in *U*, having the form:

$$P_{c}\left(z\right)=\sum_{n=0}^{\infty }p_{n}z^{n},\;z\in U.$$

It is clear that $T_{\gamma }\left(0\right)=1$ and $ℜ(T_{\gamma })>0.$

TFE has been maximized by using different techniques depending on its parameter γ. This problem was discussed in [1,2] for real power index γ and in [21] for the complex power index. The authors showed that the Tsallis distribution reserves its fractional power formula, decorating with some specific log-periodic oscillations (convergence dynamics of z-logistic maps). As a result, the authors introduced a complex measure of the thermal bath heat capacity C=1/(γ−1). Thus, in general, the heat capacity becomes complex as well. In this work, CTFE approximates some special functions in a complex domain. These functions are popular in various applications.

Next, we approximate Equation (4) for some special functions. The advantageous of the approximation are: First, for recognizing target functions, the approximation technique studies how certain known functions (for example, special functions) can be approximated by a definite class of functions (for example, polynomials or rational functions) that often have desirable properties (inexpensive computation, continuity, integral and limit values, etc.). Second, the target function, call it Ψ, may be unknown; instead of a clear formula, only a set of points of the form (x,Ψ(x)) is delivered. Depending on the organization of the domain and codomain of Ψ, several methods for approximating g may be applicable. For example, if Ψ is an operation on the complex numbers, the techniques of geometric function theory can be used.

### 2.1. Bernoulli Function ${\left[z/\left(e^{z}-1\right)\right]}^{\gamma }$

**Figure 2:**
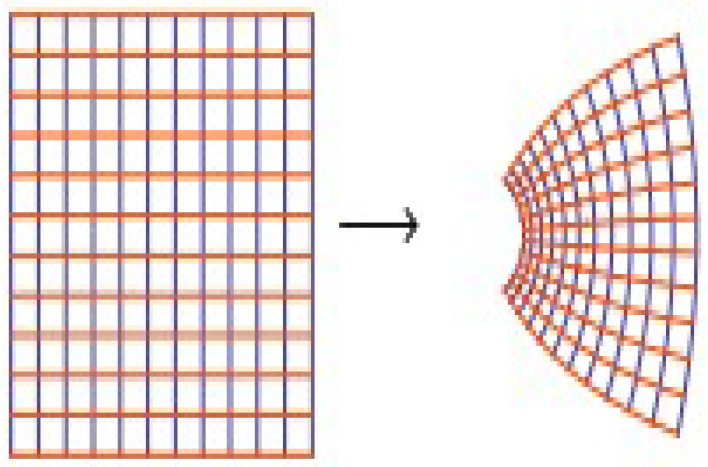
Bernoulli function $z/(e^{z}-1)$.

Mocanu [22] showed that the function $z/(e^{z}-1)$ is convex in *U* (see Figure 2).

**Figure 3:**
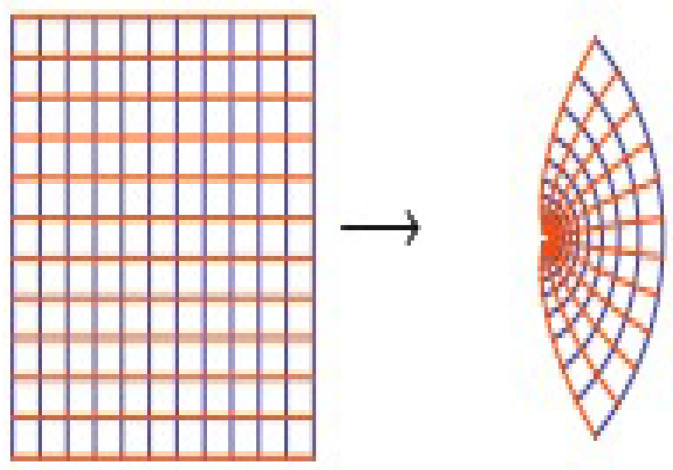
Bernoulli function ${\left[z/\left(e^{z}-1\right)\right]}^{2}$.

The function is not convex when γ≥2 (see Figure 3).

Series expansions at z=0,γ=2,… are given as follows:

(5) $$\begin{array}{c} T_{2}\left(z\right)=\;1-z+\left(5z^{2}\right)/12-z^{3}/12+z^{4}/240+O\left(z^{5}\right)\\ T_{3}\left(z\right)=1-\left(3z\right)/2+z^{2}-\left(3z^{3}\right)/8+\left(19z^{4}\right)/240+O\left(z^{5}\right)\\ T_{4}\left(z\right)=1-2z+\left(11z^{2}\right)/6-z^{3}+\left(251z^{4}\right)/720+O\left(z^{5}\right)\\ \vdots \end{array}$$

Moreover, when 0<γ<1, we have:

$$T_{0.5}\left(z\right)=1-\frac{z}{4}+\frac{z^{2}}{96}+\frac{z^{3}}{384}-\frac{z^{4}}{10240}+\ldots$$

For $\varphi \left(z\right)=\sum \varphi_{n}z^{n}$ and $\upsilon \left(z\right)=\sum \upsilon_{n}z^{n},\;\upsilon_{n}\geq 0$ for all n≥0, we have φ≪υ if and only if $|\varphi_{n}|\leq \upsilon_{n}.$ Note that this concept is called majorization coefficients.

We have the following properties (upper bounds):

**Proposition** **1.** *For CTFE approximated by Bernoulli function,*

$$T_{\gamma }\left(z\right)\ll {\left(\frac{1+z}{1-z}\right)}^{\gamma },\;\gamma >0,\gamma \neq 1.$$

**Proof.**  Let:

$$\psi \left(z,\gamma \right)={\left(\frac{1+z}{1-z}\right)}^{\gamma },\;z\in U,\gamma \neq 1.$$

Then, we obtain:

(6) $$\begin{array}{c} \psi \left(z,2\right)=1+\sum_{n=1}\left(4n\right)z^{n}=1+4z+8z^{2}+12z^{3}+16z^{4}+20z^{5}+\ldots \\ \psi \left(z,3\right)=1+\sum_{n=1}\left(2+4n^{2}\right)z^{n}=1+6z+18z^{2}+38z^{3}+\ldots .\\ \psi \left(z,4\right)=1+\sum_{n=1}\frac{1}{3}\left(8n\left(2+n^{2}\right)\right)z^{n}=1+8z+16z^{2}+24z^{3}+\ldots .\\ \vdots \end{array}$$

Furthermore, for 0<γ<1, we have:

$$\psi \left(z,0.5\right)=1+z+\frac{z^{2}}{2}+\frac{z^{3}}{2}+\frac{3z^{4}}{8}+\frac{3z^{5}}{8}+\ldots .$$

Comparing Equation (5) and Equation (6), we conclude that $T_{\gamma }\left(z\right)$ is majorized by the function ${\left(\frac{1+z}{1-z}\right)}^{\gamma }$ for all γ≠1. ☐

**Proposition** **2.** *For CTFE approximated by Bernoulli function, there is a probability measure μ on ${\left(\partial U\right)}^{2},$ for all γ>1.*

**Proof.**  Let t,τ∈∂U; then, we have:

(7) $$\begin{array}{cc} {\left(\frac{1+tz}{1+\tau z}\right)}^{\gamma } & =\frac{{\left(1+tz\right)}^{\gamma }}{1+\tau z}.\frac{1}{{\left(1+\tau z\right)}^{\gamma -1}}\\ & \ll \frac{{\left(1+z\right)}^{\gamma }}{1-z}.\frac{1}{{\left(1-z\right)}^{\gamma -1}}\\ & ={\left(\frac{1+z}{1-z}\right)}^{\gamma },\;\gamma >1. \end{array}$$

In view of Theorem 1.11 in [23], the ${\left(\frac{1+tz}{1+\tau z}\right)}^{\gamma }$ admits a probability measure μ in ${\left(\partial U\right)}^{2}$ satisfying:

$$f\left(z\right)=\int_{{\left(\partial U\right)}^{2}}{\left(\frac{1+tz}{1+\tau z}\right)}^{\gamma }d\mu \left(t,\tau \right),\;z\in U.$$

Then, by virtue of Proposition 1, there is a constant λ (diffusion constant) such that:

$$\int_{{\left(\partial U\right)}^{2}}{\left(\frac{1+tz}{1+\tau z}\right)}^{\gamma }d\mu \left(t,\tau \right)=\lambda \int_{{\left(\partial U\right)}^{2}}{\left(\frac{tz}{e^{\tau z}-1}\right)}^{\gamma }d\mu \left(t,\tau \right),\;z\in U.$$

This completes the proof. ☐

### 2.2. Gaussian Function Φ(a,c;z)

The function Φ(a,c;z) is defined by the series:

$$\Phi \left(a,c;z\right)=\frac{\Gamma (c)}{\Gamma (a)}\sum_{n=0}^{\infty }\frac{\Gamma (a+n)}{\Gamma (c+n)}\frac{z^{n}}{n!}.$$

A special case of this function is $\Phi \left(a,a;z\right)=e^{z}.$ We consider CTFE approximated by $e^{-\gamma z}.$ Clearly, we have the following results:

**Proposition** **3.** *For CTFE approximated by $e^{-\gamma z}$:*

$$T_{\gamma }\left(z\right)\ll \Phi \left(a,c;z\right),$$

$$\left(\gamma >0,\gamma \neq 1,\;\;ℜa>1,ℜc>1\right).$$

**Proposition** **4.** *For CTFE approximated by $e^{-\gamma z}$, there is a probability measure μ on [0, 1].*

**Proof.**  In view of Equation (1.2-8) in [24], there is a probability measure on [0, 1] such that:

$$\Phi \left(a,c;z\right)=\frac{\Gamma (c)}{\Gamma (a)\Gamma (c-a)}\int_{0}^{1}\tau^{a-1}{\left(1-\tau \right)}^{c-a-1}e^{\tau z}dt=\int_{0}^{1}e^{\tau z}d\mu$$

$$d\mu \left(\tau \right)=\frac{\Gamma (c)}{\Gamma (a)\Gamma (c-a)}\frac{\tau^{a-1}}{{\left(1-\tau \right)}^{c-a-1}}dt.$$

By Proposition 3, we have the desired assertion. ☐

### 2.3. Fractional Sigmoid Function FSF

**Figure 4:**
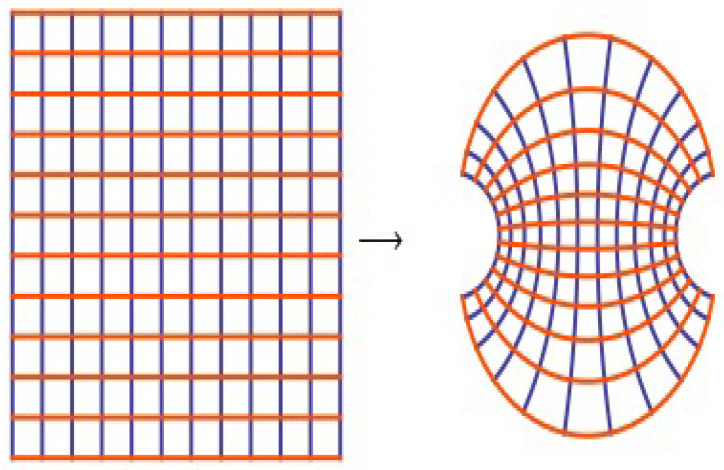
Sigmoid function $\frac{2}{1+e^{-\gamma z}},\gamma =2$.

CTFE can be approximated by FSF. In our investigation, we focus on the type of function, which is analytic in *U*. We suggest the function (see Figure 4):

(8) $$T_{\gamma }\left(z\right)=\frac{2}{1+e^{-\gamma z}},\;\gamma \neq 1,z\in U.$$

The expansion CTFE are given as follows:

(9) $$\begin{array}{c} T_{2}\left(z\right)=1+z-\frac{z^{3}}{3}+\frac{2z^{5}}{15}-\frac{17z^{7}}{315}+O\left(z^{9}\right)\\ T_{3}\left(z\right)=1+\frac{3z}{2}-\frac{9z^{3}}{8}+\frac{81z^{5}}{80}-\frac{1413z^{7}}{4480}+O\left(z^{9}\right)\\ T_{4}\left(z\right)=1+2z-\frac{8}{3}z^{3}+\frac{64}{15}z^{5}-\frac{217}{315}z^{7}+O\left(z^{9}\right)\\ \vdots \end{array}$$

For sufficient values of *a* and c, CTFE approximated by FSF can be majorized by Φ(a,c;z).

## 3. Complex-Valued Neural Networks

CNNs are a necessary extension of the analysis of real-valued neural networks. CNNs are networks that utilize complex-valued variables and parameters, effectively distributing in this style with complex-valued information. They are very well matched with wave phenomena, and they are suitable for the procedures connected with complex altitude [25]. The have been used for a long list of applications, essentially in learning tasks, loss function, cost function, utility function and combinatorial optimization.

In CNNs, the neurons in each layer are systematized as a three-dimensional array rather than as a vector in ANNs (artificial neural networks). The first two dimensions are titled spatial, and the third is a partition to networks. The CNN system charts three ideologies characteristic of natural systems: locality, sharing and pooling.

The locality behavior is the information that neurons depend only on their neighbors, rather than on far away neurons. Sharing is the limitation that various pi neurons should undergo the same processing. It is challenging that an affine layer follows locality, and sharing results in a convolution layer. Pooling is used to indicate invariance to small translations. A pooling layer does so by splitting each input channel into patches and replacing each patch with a single representative assessment in the output layer.

Suppose the CNN is delivered by *n* fully connected in a Hopfield-like net. The output is given by a complex number for each neuron:

$$Z=\{z_{1},\ldots ,z_{n}\}\subset U.$$

**Figure 5:**
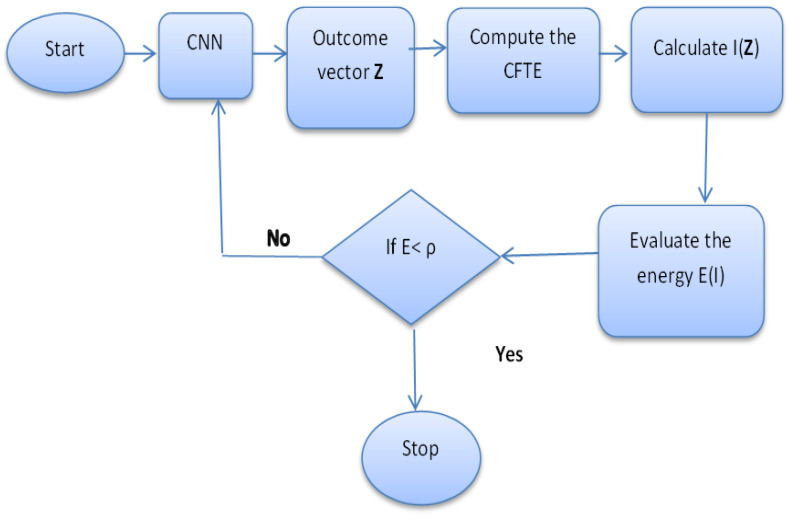
The algorithm of using CTFE in CNNs.

Thus, the network state (information of the net) $I_{\gamma }\left(z_{k}\right),\;k=1,\ldots ,n$ is a complex vector. In this work, we shall use the total information, which is given by the relation:

(10) $$I_{\gamma }\left(z\right)=\sum_{k=1}^{n}\;\frac{T_{\gamma }\left(z_{k}\right)}{\gamma -1},\;\gamma \neq 1,\;z\in Z,$$

where $T_{\gamma }$ is approximated by Equation (9). Therefore, a large amount of information can be realized from both theoretical study and numerical computations from $T_{\gamma }\left(z\right).$ The stability of Equation (10) is given by the energy equation:

(11) $$E_{\gamma }=\frac{I_{\gamma }\left(z\right){\bar{I}}_{\gamma }\left(z\right)}{n},$$

where ${\bar{I}}_{\gamma }\left(z\right)$ is the conjugate of $I_{\gamma }\left(z\right).$ The energy provides a tool for studying the dynamics of CNNs. Figure 5 shows the steps of finding the energy. The minimum energy is bounded by the value ρ, which is suggested during the training of CNN.

It has been shown by experiences, for a CNN of four neurons, that the minimum energy is satisfying Equation (11) for the output on ∂U as follows:

Z={i,−i,1,−1}.

The energy $E_{\gamma }$ is equal to one for all values γ>2; while the energy is increasing for outcomes inside the unit disk U. For example, the output set:

$$Z=\{\frac{1+i}{2},\frac{1-i}{2},\frac{i}{2},-\frac{i}{2}\}$$

has energy $E_{\gamma }>1$, for different values of γ.

### Numerical Examples

Let Z={i,−i,1,−1} be the outcome set of CNN. To apply our algorithm, we pursue the following steps:

Step 1. Calculate $T_{\gamma },\;\gamma >2$ from Equation (8) as follows: for γ=3, we have:

$$\begin{array}{c} T_{3}\left(i\right)=\frac{2}{1+e^{-3i}}=1+14.1\;i,\;T_{3}\left(-i\right)=\frac{2}{1+e^{3i}}=1-14.1\;i,\\ T_{3}\left(1\right)=\frac{2}{1+e^{-3}}=1.9,\;T_{3}\left(-1\right)=\frac{2}{1+e^{3}}=0.094; \end{array}$$

Step 2. Compute the total information by using Equation (10):

$$I_{3}\left(z\right)=\sum_{k=1}^{4}\;\frac{T_{3}\left(z_{k}\right)}{3-1}\approx 2.$$

Step 3. Estimate the energy of CNN by applying Equation (11):

$$E_{3}=\frac{I_{3}\left(z\right){\bar{I}}_{3}\left(z\right)}{4}=1.$$

**Remark** **1.** *One can show that for all γ>2, the estimate energy for the set Z={i,−i,1,−1} is equal to one. The algorithm will stop at the value ρ, which was given previously. In our example, we consider ρ=1 for all $z\in \bar{U}.$*

*Moreover, to estimate the energy of the outcomes set $Z=\{\frac{1+i}{2},\frac{1-i}{2},\frac{i}{2},-\frac{i}{2}\},$ we follow the above steps:*

$$T_{3}\left(z\right)=5.6,\;I_{3}\left(z\right)=2.8,\;E_{3}=1.96.$$

*Comparing with ρ=1, the CNN needs more training.*

**Remark** **2.** *Comparing with the complex Shannon entropy [18], we obtain the following values for the set Z={i,−i,1,−1}:*

H(i)≈1.0010005−0.999999499i,H(−i)≈1.0010005+0.999999499i,H(1)=0,H(−1)=1.

*This implies total information I(z)=3. Consequently, we have E=9/4=2.25>1.*

## 4. Discussion

- Equation (10) refers to the amount of information in the complex system, which is given in the CNN. The advantage is that CNN does not depend on the number of neurons to get full training of the system (see [11,12,13,14,15,26]). Furthermore, the complex value of the output converges to the stability state faster than the real value. All the complex value outputs are given in the open unit disk U, where |z|<1 (see [16]). In this case, we may use the properties of geometry function theory (GFT). For example, the sigmoid function of the complex value is studied widely in view of GFT. The convexity and other geometric representations of this function have been studied by many authors (see [27]).
- The parameter γ from $I_{\gamma }$ is: the simplest non-trivial perturbation of any unperturbed complex system; the complex system (CNN) in which obvious necessary and sufficient conditions are recognized for a small divisor problem is stable.
- The output may cause a complex-valued function incited by the set Z. In this situation, the stability comes from the first derivative of I(z) with respect to *z*. This type of stability is called Lyapunov stability. At a fixed point $z_{0}$:
  $$I_{\gamma }^{\prime }\left(z_{0}\right)=\frac{d}{dz}I_{\gamma }\left(z_{0}\right)=2z_{0}.$$
  At a periodic point $z_{0}$ of period *℘*, the first derivative of a function:
  $${\left(I_{\gamma }^{℘}\right)}^{\prime }\left(z_{0}\right)=\frac{d}{dz}I_{\gamma }^{℘}\left(z_{0}\right)=\prod_{i=0}^{℘-1}I_{\gamma }^{\prime }\left(z_{i}\right)=2^{℘}\prod_{i=0}^{℘-1}z_{i}=\lambda$$
  is usually given by λ and represented by the multiplier or the Lyapunov characteristic number. It applies to checking the stability of periodic points, as well as fixed points (λ=0).
- At a non-periodic point, the derivative, $z_{n}^{\prime },$ can be iterated by:
  $$z_{0}^{\prime }=1;\;z_{n}^{\prime }=2\times z_{n-1}\times z_{n-1}^{\prime }.$$
- The above derivative can be replaced by any derivative for a complex variable z∈C such as the Schwarzian derivative. We may suggest this as a future work.
- Derivative with respect to γ (parametric derivative): This type of derivative is called the distance estimation method. In this case, CNN has one output in the set Z, and it is fixed. Therefore, we suggest to use the parameter plane collecting information. This occurs as follows: On the parameter plane: γ is a variable, and $z_{0}=0$ is constant. The first derivative of $I_{\gamma }^{n}\left(z_{0}\right)$ with respect to γ is given by the relation:
  $$z_{n}^{\prime }=\frac{d}{d\gamma }I_{\gamma }^{n}\left(z_{0}\right).$$
  This derivative can be defined by the following iteration:
  $$z_{0}^{\prime }=\frac{d}{d\gamma }I_{\gamma }^{0}\left(z_{0}\right)=1$$
  and then replacing at every consecutive step:
  $$z_{n+1}^{\prime }=\frac{d}{d\gamma }I_{\gamma }^{n+1}\left(z_{0}\right)=2\cdot I_{\gamma }^{n}\left(z\right)\cdot \frac{d}{d\gamma }I_{\gamma }^{n}\left(z_{0}\right)+1=2\cdot z_{n}\cdot z_{n}^{\prime }+1.$$

## 5. Conclusions and Future Research

In the present paper, we have been applying the model of complex probability to Tsallis’ entropy. Henceforth, we established a fitted connection between the new model and the classical FTE. Therefore, we developed the theory of information. As an application, we made a generalization of CNNs; its result implied minimization of the energy in this complex system. The aid of extending FTE leads to very stimulating and successful consequences and outcomes illustrated in this work. Therefore, we are calling this original and beneficial new study in applied mathematics and analytics: “the theory of complex information”.

It is intended that additional development of this original study will be done in subsequent work such as convergence, convexity and concavity. It is proposed that in future research studies, the novel planned analytic method will be elaborated more, and the complex probability model, as well as extensive and various sets of stochastic processes will be applied.

## Abbreviations

The following abbreviations are used in this manuscript:

γ	parameter
λ	diffusion constant
*z*	complex number
$P_{c}$	complex probability
$S_{R}$	real set of events
$S_{M}$	imaginary set of events
$P_{r}$	probability in the real set
$P_{m}$	probability in the imaginary set
*U*	the open unit disk
${\left|z\right|}^{2}$	the degree of our knowledge of the random experiment; it is the square of the norm of *z*
$T_{\gamma }\left[P_{c}\right]$	CFTE
$ℜ(T_{\gamma })$	the real part of CFTE
Φ(a,c;z)	Gaussian function
Γ(.)	gamma function
$I_{\gamma }$	total information
$E_{\gamma }$	the energy
ρ	the upper bound of energy
